# Identifying common factors of functioning, participation and environment amongst adults requiring specialist oral health care using the International Classification of Functioning, disability and health

**DOI:** 10.1371/journal.pone.0199781

**Published:** 2018-07-03

**Authors:** Alison Dougall, Francisca Martinez Pereira, Gustavo Molina, Caroline Eschevins, Blánaid Daly, Denise Faulks

**Affiliations:** 1 Dublin Dental University Hospital, Trinity College, Dublin, Ireland; 2 University of Santiago de Compostela, Santiago de Compostela, Spain; 3 Escuela de Odontologia, Universidad Catolica de Cordoba, Cordoba, Argentina; 4 Université Clermont Auvergne, CROC EA4847, Clermont Ferrand, France; 5 Division of Population and Public Health, Dental Institute, King’s College, London, United Kingdom; 6 CHU Clermont-Ferrand, Service d’Odontologie, Clermont Ferrand, France; University of Illinois at Urbana-Champaign, UNITED STATES

## Abstract

**Introduction:**

Persons unable to access oral health care in the conventional primary health care setting suffer from inequalities in oral health, particularly in terms of unmet dental need. The International Classification of Functioning, disability and health (ICF) is designed to look beyond medical diagnosis and to describe individuals or populations in terms of their ability to function and participate in a social environment. The objective of the study was to describe an adult population requiring specialist oral health care using the ICF and to identify common factors of functioning, participation and environmental context.

**Method:**

The ICF Checklist for Oral Health was completed for 246 participants from five specialist dental services in five countries (mean age 36 ±16.44 years; 16–92). ‘Developmental disability’ and ‘Medically compromised’ groups were identified (72% and 28%).

**Results:**

Participants presented with oral disease (92%) and dysfunction (66% impaired chewing). 33 ICF items were affected in over 50% of participants in both groups. Impaired body functions included ‘ingestion functions’, ‘energy and drive functions’ and ‘emotional functions’. Participation was restricted for “Acquiring, keeping and terminating a job”, “Intimate relationships”, “Handling stress and psychological demands”, “Economic self-sufficiency”, “Carrying out a daily routine”, “Recreation and leisure”, “Community life” and “Looking after one’s health”. In the environment domain, “Support and relationships” and “Attitudes” were rated as facilitators. Environmental barriers reported for over 25% of the whole group were related to “Services, systems and policies” including, health, social security, general support, transportation, and labour and employment.

**Discussion and perspectives:**

Common aspects of functioning, participation and environment were found amongst a heterogeneous population of adults attending specialist dental services, alongside poor oral health and function. The ICF may be used to describe populations that suffer inequality in oral health in order to develop services that effectively target those in need of additional means.

## Introduction

Special care dentistry is defined by the International Association for Disability and Oral Health as dentistry for individuals with a disability or activity restriction that directly or indirectly affects their oral health, within the personal and environmental context of the individual [1]. The majority of these patients will receive care in the primary health care sector and a minority with more complex needs will require specialist care [2]. Persons with special health care needs have been shown to suffer severe inequality in oral health in relation to poor oral hygiene, substantially higher levels of periodontal disease, and higher prevalence of untreated decay and extracted teeth in comparison with the general population [3]. Groups for whom the primary health care practitioner may not be able to provide high quality dental treatment include persons unable to cooperate with treatment due to neuromotor or cognitive disability, or persons with complex medical issues that require adaptive measures. Traditionally, these populations have been defined using medical criteria–specifically medical diagnosis. However, it is increasingly recognised that two persons with the same diagnosis will not have the same needs. For example, one person with Down’s syndrome may have virtually no problems accessing or receiving dental care in the conventional setting, whilst another may be unable to receive treatment without sedation or general anaesthesia.

The International Classification of Functioning, disability and health (ICF) is designed to look beyond medical diagnosis and to describe individuals or populations in terms of their ability to function and participate in a social environment [4]. For this reason it is an appropriate tool for the description of the populations requiring specialist care in dentistry. The aim of collecting data to describe populations that suffer inequality in oral health is to develop services that effectively target those in need of additional means. The ICF has previously been used to describe a paediatric population requiring specialist dental care, but has not yet been used in this context with adults.

In order to integrate the philosophy of the ICF into the discipline of dentistry, it is necessary to develop an ICF Core Set for Oral Health (a subset of ICF items for use in the specific field of oral health). ICF Core Sets have been produced for a large number of health domains to date and the WHO provides a detailed methodology to guide this process [5]. Empirical data is required for the development of such an ICF Core Set in Oral Health. This universal tool would clarify issues of functioning and environment with relation to the ability to maintain oral health and access care.

### Objectives

1) To describe an adult population requiring specialist oral health care using the International Classification of Functioning, disability and health.
2) To identify common factors of functioning, participation and environmental context within this population, and between groups with developmental disability and those with complex medical history.
3) To collect the data necessary to inform the process of ICF Core Set development for oral health.

## Materials and methods

The methodology used in this empirical, cross-sectional study followed that developed by the ICF Research Branch of the WHO Collaborating Centre for the Family of International Classifications (DIMDI, Germany) in partnership with the World Health Organisation Classification, Terminology and Standards group (CTS) [5,6].

### The questionnaire

The ICF Checklist [7] was modified to give an ICF Checklist for Oral Health. Items specific to oral health but that did not appear in the original WHO Checklist were added using a previous list established by Faulks & Hennequin (2006) [8] and from the results of a survey of professional opinion of oral health [9]. An additional question was added to the general medical section of the checklist regarding perception of oral health [10], as it has been proposed that oral and general health must be regarded as separate constructs [11,12].

The resulting ICF Checklist for Oral Health recorded:

- demographic information
- medical diagnoses using the International Classification of Diseases (ICD-10: World Health Organisation, 1992–1994) [13]
- dental diagnoses using the International Classification of Diseases–Application to Dentistry and Stomatology (ICD-DA: World Health Organisation, 1995) [14]; the DMFT Index [15], which gives a composite score for decayed teeth (D), teeth missing due to caries (M), and filled teeth (F); and the number of posterior functional dental units (the number of pairs of posterior teeth in contact on closing mouth, excluding third molars so a maximum of 8) [16].
- information regarding other health related issues (e.g. use of medication, need for assistance in daily living)
- self-rated patient and/or caregiver subjective perception of physical, mental and oral health on a five point Likert scale from ‘Very good’ to ‘Very poor’
- presence or absence of an impairment for a list of items from the *Body Functions* component of the ICF (44 items)
- presence or absence of an impairment for a list of oral structures from the *Body Structures* component of the ICF (7 items)
- presence or absence of restriction in participation for a list of items from the *Activities and Participation* component of the ICF (44 items)
- presence of a barrier or facilitating factor (facilitator) for a list of items from the *Environmental* component of the ICF (23 items)
- other relevant contextual information completed free-hand by the investigator (for example, personal factors such as lack of immediate family, immigration status, life events).

The ICF Checklist for Oral Health was produced in English, French, and Spanish, using pre-existing WHO translations of ICF items.

### Investigator training

The investigators undertook an on-site training programme regarding the use of the ICF and on the use of the ICF Checklist for Oral Health in particular. Training included case studies, item by item examples, and peer review of questionnaire completion with role-playing to ensure consensus and consistency.

### Data collection

Data was collected in five specialist dental clinics in France, Argentina, Spain, Ireland, and the UK from May 2015 to July 2016. A convenience sample was constructed by recruiting all patients fulfilling the inclusion criteria consecutively, in order of presentation to the service, on the days when the investigator was present. The ICF Checklist for Oral Health was completed by the investigator based on information in the medical/dental records, direct observation of the patient and from a structured interview with the patient and/or primary carer. Before interviewing the patient and/ or caregiver, the medical and dental files of the patient were studied and all relevant data pre-completed. Patient observation took place during the clinical visit. The interview was then used by the investigating clinician to complete missing data. The interview could take place at distance from initial inclusion in the study. The ICF Checklist for Oral Health took approximately 30 minutes to complete for each participant.

### Ethical considerations

The relevant local ethical committee for each study centre approved the protocol, the patient information letter and consent form (Comité d’Ethique des Centres d’Investigation Clinique de l’Inter-région Rhône-Alpes-Auvergne, France; Comité de Bioética de la Universidad de Santiago de Compostela, Spain; Joint Research Ethics Committee, School of Dental Science, Trinity College Dublin, Ireland; Comité Institucional De Ética De La Investigacion En Salud, Clínica Universitaria Privada Reina Fabiola, Fundación para el Progreso de la Universidad Católica de Córdoba, Argentina; Health Research Authority London–Harrow research Ethics Committee, and Research and Innovation Office, Kings College Hospital NHS, London England). Data collection was anonymous. The information letter and consent forms were adapted to local requirements but in all centres patients were informed of the objectives of the study verbally and in writing. Signed consent for participation was acquired from the participant and/or their legal guardian. The study was declared to the relevant national committee for data protection.

### The study population

The following inclusion criteria applied:

i) Patient 16 years of age or above on the day of data collection.
ii) Patient referred to a special care dental unit or other specialist dental service because of a medical condition or other problem rendering dental care in mainstream services difficult or inappropriate.
iii) Patient with a signed consent form. Informed consent was sought from patients and/or their legal representatives for anonymous data collection and analysis.

The study population was not intended to be representative. The study was designed to recruit as heterogeneous a population as possible in order to reflect the wide scope of special care dentistry and the aim of data collection in terms of the ICF was to be as exhaustive as possible. Taking into account previous publications following the ICF Core Set methodology, the aim was to record data for 240 patients.

### Data entry and analysis

Central, double, data entry was performed using Microsoft Excel^®^. Descriptive statistics were used to describe the study population and to examine the frequency of problems recorded by the ICF Checklist for Oral Health. For the ICF components Body Functions, Body Structures and Activities and Participation absolute frequencies and relative frequencies (prevalence) of impairment/limitation in the study population were calculated. For Environmental Factors, absolute frequencies and relative frequencies (prevalence) of items entered as either a barrier or facilitator were reported.

Traditionally in dentistry, patients requiring specialist care are divided into those with developmental or intellectual disability, and those with complex medical history. For analysis, the study population was therefore divided into two main groups according to principal medical diagnosis using the ICD-10. This division was designed to confirm the heterogeneity of the patient population. Patients were considered as having a ‘Developmental Disability’ if their principal medical diagnosis was within chapters F, G or Q of the ICD-10, and was present from birth or with onset during the developmental period (F: Mental and behavioural disorders; G: Diseases of the nervous system; Q: Congenital malformation, deformation and chromosomal abnormalities). Patients with any other principal medical diagnosis were considered to be ‘Medically compromised’. All patients could, of course, present other concurrent, secondary medical conditions. The frequency with which an item was reported for each of these patient groups was compared using a χ^2^ test. In addition, differences between countries were analysed for the Environmental factors, as it was assumed that local context would have an impact on service provision.

## Results

### Study population

The demographic data for the 246 participants are given in Table 1. The majority of patients were male (57.7%) and the mean age was 35.89 ± 16.44 years with an age range of 16 to 92 years.

**Table 1:** Description of study population.

	ALL	Developmental Disability	Medically Compromised	difference between groups
**Number of participants (%)**	**246 (100%)**	**178 (72.4%)**	**68 (27.6%)**	
Female sex	102/241 (42.3%)	76/177 (42.9%)	26/64 (40.6%)	^a^ ns
Age mean ± SD years	35.89 ± 16.44	30.56 ± 10.31	49.85 ± 20.87	^b^ p<0.001
Age range years	16 to 92	16 to 57	18 to 92	
**Country**	**N = 246 patients**	**N = 178**	**N = 68**	
France	61 (24.8%)	56 (31.5%)	5 (7.4%)	
Spain	60 (24.4%)	57 (32.0%)	3 (4.4%)	
Argentina	61 (24.8%)	54 (30.3%)	7 (10.3%)	
Ireland	59 (24.0%)	9 (5.1%)	50 (73.5%)	
England	5 (2.0%)	2 (1.1%)	3 (4.4%)	
**Type of residence (> one option possible)**	**N = 246 patients**	**N = 178**	**N = 68**	
Own home	127 (51.6%)	72 (40.4%)	55 (80.9%)	^a^ p<0.001
Caregiver’s home	94 (38.2%)	83 (46.6%)	11 (16.2%)	^a^ p<0.001
Institution	52 (21.1%)	53 (28.8%)	2 (2.9%)	^a^ p<0.001
**Occupation**	**N = 243 patients**	**N = 177**	**N = 66**	
Active (Mainstream, protected or unpaid employment or student)	93 (38.3%)	72 (40.7%)	22 (33.3%)	^a^ ns
Unemployed due to medical condition or incapacity	104 (42.8%)	92 (52.0%)	24 (36.4%)	^a^ p<0.05
Retired	23 (9.5%)	4 (2.25%)	19 (28.78%)	na
Homemaker or other	20 (8.2%)	19 (10.7%)	1 (1.51%)	na
**Assistance / therapy**	**N = 246 patients**	**N = 178**	**N = 68**	
Regular medication	170 (69.1%)	113 (63.5%)	57 (83.8%)	^a^ p<0.01
Assistive devices	146 (59.3%)	101 (56.7%)	45 (66.2%)	^a^ ns
Assistance for daily living	185 (75.2%)	152 (85.4%)	33 (48.5%)	^a^ p<0.001
Paramedical therapy	124 (50.4%)	97 (54.5%)	27 (39.7%)	^a^ p<0.05

^a^ χ^2^ test between groups

^b^ Student’s t-test between groups

The ‘Developmental disability’ group represented 72.4% of the study population. Patients in the ‘Medically compromised’ group were significantly more likely to be older, live in their own home and take regular medication. Patients in the ‘Developmental disability’ group were significantly more likely to live in a caregiver’s home or an institution, to be unemployed due to medical condition or incapacity, to need assistance for daily living and to benefit from paramedical therapy.

The five countries participating in the study were equally represented apart from England, as the investigator at this centre moved abroad during the study period and inclusions were discontinued. The majority of patients from France, Spain and Argentina were in the ‘Developmental disability’ group and the majority of patients from Ireland and England were in the ‘Medically compromised’ group.

### Medical and dental health

All participants presented with at least one medical diagnosis and 483 diagnoses were reported in total for the study population. Diagnosed medical conditions reported by over 5% of the participants are given in Table 2. Almost half of participants presented with a mental or behavioural disorder and/or a disease of the nervous system. A quarter of participants had a congenital malformation, deformation or chromosomal abnormality.

**Table 2:** Description of the medical conditions recorded.

	ICD diagnosis reported by > 5% of patients	Number times a diagnosis reported (N = 427) (> one diagnosis per patient possible)	% of patients with diagnosis (N = 246 patients)
**D50-89**	**Non-malignant neoplasms and haematological disorders**	**18**	**7.3%**
Of which	Coagulation defects and haemorrhagic conditions	15	6.1%
**E00-E90**	**Endocrine, nutritional and metabolic disease**	**43**	**17.5%**
**F00-F90**	**Mental and behavioural disorders**	**121**	**49.2%**
Of which	Mental retardation	70	28.5%
	Disorders of psychological development	31	12.6%
**G00-G99**	**Diseases of the nervous system**	**116**	**47.2%**
Of which	Episodic and paroxysmal disorders	48	19.5%
	Cerebral palsy and other paralytic syndromes	46	18.7%
**H00-H95**	**Diseases of the eye and ear**	**24**	**9.8%**
Of which	Disorders of the eye	19	7.7%
**I00-I99**	**Diseases of the circulatory system**	**29**	**11.8%**
**M00-M99**	**Diseases of the musculoskeletal system**	**14**	**5.7%**
**Q00-Q99**	**Congenital malformation, deformation and chromosomal abnormalities**	**62**	**25.2%**
Of which	Chromosomal abnormalities including Down syndrome	38	15.5%

In terms of objective dental health (Table 3), DMFT was high for all groups. Participants in the ‘Medically compromised’ group had a significantly higher overall DMFT and a higher mean number of missing teeth than those in the ‘Developmental disability’ group. Over 90% of patients presented with a dental ICD-10 diagnosis and those in the ‘Developmental disability’ group were significantly more likely to present with any oral diagnosis, with gingivitis and periodontitis, with a malocclusion and with bruxism. In the ‘Developmental disability’ group 70% of patients presented with gingivitis or periodontal disease compared to 45% in the ‘Medically compromised’ group. Almost half of all patients presented with dental caries and over 20% had less than five functional dental pairs, a marker of masticatory deficiency.

**Table 3:** Description of oral health of participants.

		ALL	Developmental disability	Medically compromised	
**DMFT**		**N = 244**	**N = 177**	**N = 67**	t-test
	Mean ± SD	11.02 ± 8.25	9.43 ± 7.07	15.22 ± 9.64	p<0.001
	Max; Min	28; 0	28; 0	29; 0	
Decayed	Mean ± SD	2.11 ± 3.10	2.04 ± 3.12	2.28 ± 3.08	ns
	Max Min	19; 0	19; 0	12; 0	
Missing	Mean ± SD	4.77 ± 6.8	3.38 ± 4.88	8.44 ± 9.53	p<0.001
	Max Min	28; 0	28; 0	28; 0	
Filled	Mean ± SD	4.14 ± 4.21	4.00 ± 4.17	4.49 ± 4.28	ns
	Max Min	17 ; 0	17 ; 0	16; 0	
**Functional pairs**		**N = 243**	**N = 177**	**N = 66**	χ^2^ test between groups
Less than 5 pairs	n (%)	55 (22.6%)	36 (20.3%)	19 (28.8%)	ns
5 to 7 pairs	n (%)	93 (38.3%)	72 (40.7%)	21 (31.8%)	
More than 7 pairs	n (%)	95 (39.1%)	69 (39.0%)	26 (39.4%)	
**Presenting with an ICD-DA diagnosis**		**N = 245**	**N = 178**	**N = 67**	χ^2^ test between groups
Any diagnosis	n (%)	226 (92.2%)	168 (94.4%)	58 (86.6%)	p<0.05
Gingivitis and periodontal disease	n (%)	155 (63.3%)	125 (70.2%)	30 (44.8%)	p<0.001
Dental caries	n (%)	121 (49.4%)	84 (47.2%)	37 (55.2%)	ns
Dentofacial anomalies including malocclusion	n (%)	81 (33.1%)	76 (42.7%)	5 (7.5%)	p<0.001
Bruxism	n (%)	39 (15.9%)	34 (19.1%)	5 (7.5%)	p<0.05
Disturbances of salivary secretion	n (%)	15 (6.1%)	13 (7.3%)	2 (3.0%)	na
Mycosis	n (%)	8 (3.3%)	1 (0.6%)	7 (10.5%)	na

### Self-rating of general, mental and oral health

The results of the subjective qualifiers of health are given in Table 4. Patients in the ‘medically compromised’ group were significantly more likely to consider their medical and oral health as ‘moderate, poor or very poor’ when compared to the group with ‘Developmental disability’. No significant difference was found between groups for subjective perception of mental health. The group with ‘Developmental disability’ were generally considered by themselves or their caregivers to be in good or very good general health (83.1%).

**Table 4:** Self-rating of physical, mental and oral health.

		ALL	Developmental disability	Medically compromised	χ^2^ test between groups
		**n = 246**	**n = 178**	**n = 68**	
**Physical health**	Very good	31.3%	38.8%	11.8%	
	Good	41.1%	44.4%	32.4%	
	Moderate	19.9%	14.0%	35.3%	
	Poor	6.9%	1.7%	20.6%	
	Very poor	0.8%	1.1%	0%	
	Moderate+Poor+Very Poor	27.6%	16.9%	55.9%	p<0.001
**Mental Health**	Very good	21.5%	20.8%	23.5%	
	Good	42.7%	44.9%	36.8%	
	Moderate	26.8%	27.5%	25.0%	
	Poor	7.3%	5.6%	11.8%	
	Very poor	1.6%	1.1%	2.9%	
	Moderate+Poor+Very Poor	35.8%	34.3%	39.7%	ns
**Oral Health**	Very good	12.2%	14.0%	7.4%	
	Good	39.4%	42.7%	30.9%	
	Moderate	28.5%	28.1%	29.4%	
	Poor	13.0%	10.1%	20.6%	
	Very poor	6.9%	5.1%	11.8%	
	Moderate+Poor+Very Poor	48.4%	43.3%	61.8%	p<0.01

### ICF items

#### Body functions

The items in the Body functions domain of the ICF that were impaired in over 50% of the study population are shown in Table 5. There was a significant difference between the ‘Developmental disability’ group and the ‘Medically compromised’ group for all items. Thirteen of the 19 Body functions items impaired in over 50% of the study population belong to the ICF Chapter “Mental functions” and were predominantly impaired in the ‘Developmental disability’ group. However, two Mental function items–“Energy and drive functions” and “Emotional functions” were also impaired in at least half of the participants in the ‘Medically compromised’ group. “Muscle tone function” was the only other item impaired in both groups for over 50% of participants. “Weight maintenance function” was the only item in this domain that was impaired for over half the participants in the ‘Medically compromised’ group, but not in the ‘Developmental disability’ group.

**Table 5:** Frequency of impairment in ICF categories of the extended ICF checklist for oral health for “Body functions”.

Body functions Cited by ≥50% of patients in any group		ALL			Developmental disability			Medically compromised			
		N response	n impairment	%	N response	n impairment	%	N response	n impairment	%	χ^2^ test between groups
**b130**	Energy and drive functions	246	191	**78.1%**	178	154	**86.5%**	68	38	**55.9%**	<0.001
**b152**	Emotional functions	246	188	**76.4%**	178	154	**86.5%**	68	34	**50.0%**	<0.001
**b122**	Global psychosocial functions	245	184	**75.1%**	178	160	**89.9%**	*67*	*24*	*35*.*8%*	<0.001
**b164**	High-level cognitive functions	246	184	**75.1%.**	178	160	**89.9%**	*68*	*22*	*35*.*3%*	<0.001
**b147**	Psychomotor functions	246	178	**72.4%**	178	160	**89.9%**	*68*	*18*	*26*.*5%*	<0.001
**b117**	Intellectual functions	245	177	**72.2%**	178	161	**90.5%**	*67*	*16*	*23*.*9%*	<0.001
**b140**	Attention functions	246	175	**71.1%**	178	152	**85.4%**	*68*	*23*	*33*.*8%*	<0.001
**b180**	Experience of self and time functions	246	170	**69.1%**	178	148	**83.2%**	*68*	*22*	*32*.*4%*	<0.001
**b735**	Muscle tone functions	246	166	**67.5%**	178	130	**73.0%**	68	36	**52.9%**	<0.01
**b167**	Mental functions of language	246	162	**65.9%**	178	151	**84.8%**	*68*	*11*	*16*.*2%*	<0.001
**b114**	Orientation functions	245	160	**65.3%**	178	146	**82.0%**	*67*	*14*	*20*.*9%*	<0.001
**b320**	Articulation functions	246	158	**64.2%**	178	143	**80.3%**	*68*	*15*	*22*.*1%*	<0.001
**b144**	Memory functions	246	157	**63.8%**	178	135	**75.8%**	*68*	*22*	*32*.*4%*	<0.001
**b710**	Mobility of joint functions	246	147	**59.8%**	178	114	**64.0%**	*68*	*23*	*48*.*5%*	<0.001
**b110**	Consciousness functions	245	146	**59.6%**	178	134	**75.3%**	*67*	*12*	*17*.*9%*	<0.001
**b156**	Perceptual functions	246	144	**58.5%**	178	132	**74.2%**	*68*	*24*	*17*.*7%*	<0.001
**b730**	Muscle power functions	246	99	**57.7%**	178	109	**61.2%**	*68*	*23*	*48*.*5%*	<0.001
**b760**	Control of voluntary movement functions	246	138	**56.1%**	178	124	**69.7%**	*68*	*14*	*20*.*6%*	<0.001
**b765**	Involuntary movement functions	246	123	**50.0%**	178	111	**62.4%**	*68*	*12*	*17*.*7%*	<0.001
**b530**	Weight maintenance function	246	*88*	*35*.*8%*	178	*51*	*28*.*7%*	68	37	**54.4%**	<0.001

In terms of oral functions (Table 6), “Chewing function” was the only item impaired in both groups at over 50% prevalence. However, all oral functions were impaired in both groups for over 20% of the study population, with the exception of “Sucking function” in the ‘Medically compromised’ group. The two groups were significantly different with higher levels of impairment found in the ‘Development disability’ group for “Chewing”, “Manipulating food in the mouth”, “Biting” and “Sucking” functions.

#### Oral structures

The oral structures cited as impaired are listed in Table 7. Both “Structure of the teeth” and “Structure of the gums” were reported as being impaired in over 50% of participants in both the ‘Developmental disability’ and the ‘Medically compromised’ groups, and there was no significant difference between groups.

**Table 6:** Frequency of impairment in ICF categories of the extended ICF checklist for oral health for oral “Body functions”.

Body functions: Oral		ALL			Developmental disability			Medically compromised			
		N response	n impairment	%	N response	n impairment	%	N response	n impairment	%	χ^2^ test between groups
**b5102**	Chewing function	245	162	**66.1%**	177	124	**70.1%**	68	38	**55.9%**	<0.05
**b5103**	Manipulation of food in the mouth	246	147	**59.8%**	178	121	**68.0%**	68	*26*	*38*.*2%*	<0.001
**b5101**	Biting function	246	131	**53.3%**	178	106	**59.6%**	68	*25*	*36*.*8%*	<0.01
**b5105**	Swallow function	246	*90*	*36*.*6%*	178	*70*	*39*.*3%*	68	*20*	*29*.*4%*	ns
**b5100**	Sucking function	244	*79*	*32*.*4%*	177	*73*	*41*.*2%*	67	*6*	*9*.*0%*	<0.001
**b5104**	Salivation	246	*69*	*28*.*0%*	178	*55*	*30*.*9%*	68	*14*	*20*.*6%*	ns
**b250**	Taste function	246	*68*	*27*.*6%*	178	*51*	*28*.*6%*	68	*17*	*25*.*0%*	ns

**Table 7:** Frequency of impairment in ICF categories of the ICF checklist for oral health for oral “Body structures”.

Body structures: Oral		ALL			Developmental disability			Medically compromised			
		N response	n impairment	%	N response	n impairment	%	N response	n impairment	%	χ^2^ test between groups
**s3200**	Structure of the teeth	246	180	**73.2%**	178	132	**74.16**	68	48	**70.59**	ns
**s3201**	Structure of the gums	246	156	**63.4%**	178	119	**66.85**	68	37	**54.41**	ns
**s3203**	Structure of tongue	246	*52*	*21*.*1%*	178	*39*	*21*.*9%*	68	*13*	*19*.*1%*	ns
**s3202**	Structure of palate	246	*49*	*19*.*9%*	178	*40*	*22*.*5%*	68	*9*	*13*.*2%*	ns

#### Activities and participation

Restricted participation was reported for over 50% of either group for 38 items (Table 8). The items that affected over 50% of participants in both ‘Developmental disability’ and ‘Medically compromised’ groups were “Acquiring, keeping and terminating a job”, “Intimate relationships”, “Handling stress and psychological demands”, “Economic self-sufficiency”, “Carrying out a daily routine”, “Recreation and leisure”, “Community life” and “Looking after one’s health”. Restriction in all items was significantly higher in the ‘Developmental disability’ group, apart from ‘Lifting and carrying objects’.

**Table 8:** Frequency of restriction in participation in ICF categories of the extended ICF checklist for oral Health for “Activities and participation”.

Participation Items cited by ≥ 50% of any group		ALL			Developmental disability			Medically compromised			
		N response	n impairment	%	N response	n impairment	%	N response	n impairment	%	χ^2^ test between groups
**d845**	Acquiring, keeping and terminating a job	226	199	**88.1%**	172	162	**94.2%**	54	37	**68.5%**	p<0.001
**d770**	Intimate relationships	246	207	**84.2%**	178	168	**94.4%**	68	39	**57.4%**	p<0.001
**d240**	Handling stress / psychological demands	246	199	**80.9%**	178	162	**91.0%**	68	37	**54.4%**	p<0.001
**d870**	Economic self-sufficiency	234	185	**79.1%**	167	158	**94.6%**	67	27	**59.7%**	p<0.001
**d230**	Carrying out daily routine	246	193	**78.5%**	178	161	**87.1%**	68	38	**55.9%**	p<0.001
**d920**	Recreation and leisure	245	192	**78.4%**	177	153	**85.9%**	68	40	**58.8%**	p<0.001
**d220**	Undertaking multiple tasks	245	190	**77.6%**	177	159	**89.8%**	68	31	*45*.*6%*	p<0.001
**d910**	Community life	245	190	**77.6%**	177	155	**87.6%**	68	35	**51.5%**	p<0.001
**d630**	Preparing meals	246	190	**77.2%**	178	160	**89.9%**	68	30	*44*.*1%*	p<0.001
**d820**	School education	225	171	**76.0%**	175	156	**89.1%**	50	15	*30*.*0%*	p<0.001
**d620**	Acquisition of goods and services	246	182	**74.0%**	178	154	**86.5%**	68	28	*41*.*2%*	p<0.001
**d740**	Formal relationships	244	180	**73.8%**	178	157	**88.2%**	66	23	*34*.*9%*	p<0.001
**d860**	Basic economic transactions	243	178	**73.3%**	177	162	**91.5%**	66	16	*24*.*2%*	p<0.001
**d730**	Relating with strangers	245	181	**73.1%**	178	152	**85.4%**	67	27	*40*.*3%*	p<0.001
**d155**	Acquiring skills	244	178	**73.0%**	177	158	**89.3%**	68	20	*29*.*4%*	p<0.001
**d177**	Making decisions	244	178	**73.0%**	177	154	**87.0%**	68	24	*35*.*3%*	p<0.001
**d950**	Political life and citizenship	240	173	**72.1%**	173	160	**92.5%**	67	13	*19*.*4%*	p<0.001
**d175**	Solving problems	244	173	**70.9%**	177	154	**87.0%**	68	19	*27*.*9%*	p<0.001
**d720**	Complex interpersonal interactions	245	171	**69.8%**	177	151	**85.3%**	68	20	*29*.*4%*	p<0.001
**d350**	Producing body language	246	171	**69.5%**	178	153	**86.0%**	68	18	*26*.*5%*	p<0.001
**d163**	Purposeful thinking	244	169	**69.3%**	177	151	**85.3%**	68	18	*26*.*5%*	p<0.001
**d440**	Fine hand use	246	168	**68.3%**	178	146	**82.0%**	68	22	*32*.*4%*	p<0.001
**d330**	Speaking	246	168	**68.3%**	178	150	**84.3%**	68	18	*26*.*5%*	p<0.001
**d570**	Looking after one's health	245	165	**67.4%**	178	130	**73.0%**	67	35	**52.2%**	p<0.01
**d520**	Caring for body parts	246	163	**66.3%**	178	134	**75.3%**	68	29	*42*.*7%*	p<0.001
**d210**	Undertaking a single task	245	161	**65.7%**	177	142	**80.2%**	68	19	*27*.*9%*	p<0.001
**d710**	Basic interpersonal interactions	245	156	**63.7%**	178	138	**77.5%**	67	18	*26*.*9%*	p<0.001
**d130**	Copying	244	154	**63.1%**	177	140	**79.1%**	68	14	*20*.*6%*	p<0.001
**d335**	Producing nonverbal messages	246	153	**62.2%**	178	140	**78.7%**	68	13	*19*.*1%*	p<0.001
**d445**	Hand and arm use	246	144	**58.5%**	178	121	**68.0%**	68	23	*33*.*8%*	p<0.001
**d510**	Washing oneself	246	144	**58.5%**	178	118	**66.3%**	68	26	*38*.*2%*	p<0.001
**d315**	Non-verbal communication	246	139	**56.5%**	178	129	**72.5%**	68	10	*14*.*7%*	p<0.001
**d310**	Verbal communication	246	137	**55.7%**	178	127	**71.4%**	68	10	*14*.*7%*	p<0.001
**d120**	Purposeful sensing	244	132	**54.1%**	177	121	**68.4%**	68	11	*16*.*2%*	p<0.001
**d110**	Watching	245	134	**50.6%**	177	113	**63.8%**	68	11	*16*.*2%*	p<0.001
**d550**	Eating	246	124	**50.4%**	178	98	**55.1%**	68	26	*38*.*2%*	p<0.05
**d115**	Listening	244	119	*48*.*8%*	177	108	**61.0%**	68	11	*16*.*2%*	p<0.001
**d430**	Lifting and carrying objects	246	122	*49*.*6%*	178	89	**50.0%**	68	33	*48*.*5%*	ns

#### Environment

Items of the Environment domain of the ICF with an impact for over 50% of either group are shown in Table 9. Eleven items were significantly different between those in the ‘Developmental disability’ and ‘Medically compromised’ groups. Items relating to food; medication; products and technology for daily living; attitudes of health professionals; and support of health professionals impacted patients in the ‘Medically compromised’ group more frequently than those in the ‘Developmental disability’ group. Attitudes of extended family; education services systems and policies; and support of personal care providers and assistants were more frequently cited for those in the ‘Developmental Disability’ group.

Items which were considered to be environmental barriers for over 25% of the whole group were related to ‘Services, systems and policies’ including, health, social security, general support, transportation, and labour services systems and policies. Table 10 shows the Environmental factors cited by 25% or over of participants from any country, excluding England because of the small sample size. Environmental barriers were particularly strong in Argentina in terms of services, systems and policies, although the significance of this difference could not be calculated due to small numbers in certain groups.

**Table 9:** Frequency of impact of ICF categories of the extended ICF checklist for oral health for “Environment”, by patient group.

ICF items impacting >50% of any group		ALL				Developmental disability				Medically compromised				
	n	% barr*	% facil*	% impact	n	% barr*	% facil*	% impact	n	% barr*	% facil*	% impact	χ^2^ test between groups for impact
**e310**	Support of immediate family	244	9.0	**84.8**	**93.9**	176	10.2	**85.8**	**96.0**	68	5.9	**82.4**	**88.2**	p<0.05
**e410**	Attitude of immediate family	245	11.8	**80.4**	**92.2**	177	13.0	**81.4**	**94.4**	68	8.8	**77.9**	**86.8**	p<0.05
**e580**	Health services, systems and policies	245	34.7	**54.7**	**89.4**	177	40.0	48.0	**87.0**	68	23.5	**72.1**	**95.6**	na
**e570**	Social security services, systems and policies	244	29.1	**58.2**	**87.3**	176	35.2	**54.0**	**89.2**	68	13.2	**69.1**	**82.4**	ns
**e575**	General social support services, systems and policies	244	32.0	**54.1**	**86.1**	176	38.6	**50.0**	**86.6**	68	14.7	**67.7**	**79.4**	ns
**e320**	Support of friends	243	7.8	**74.9**	**82.7**	176	9.7	**72.7**	**82.4**	67	3.0	**80.6**	**83.6**	ns
**e540**	Transportation services, systems and policies	245	29.4	**51.0**	**80.4**	177	33.3	46.3	**79.7**	68	19.1	**63.2**	**82.4**	ns
**e415**	Attitude of extended family	244	12.3	**68.0**	**80.3**	176	13.6	**73.3**	**86.9**	68	8.8	**54.4**	**63.2**	p<0.001
**e455**	Attitude of health-related professionals	244	7.0	**72.5**	**79.5**	176	5.7	**69.3**	**75.0**	68	10.3	**80.9**	**91.2**	p<0.01
**e420**	Attitude of friends	245	6.9	**71.0**	**78.0**	177	8.5	**69.5**	**78.0**	68	2.9	**75.0**	**77.9**	ns
**e360**	Support of other professionals	242	7.4	**69.4**	**76.9**	175	6.3	**67.4**	**73.7**	67	10.5	**74.6**	**85.1**	ns
**e355**	Support of health professionals	242	9.1	**65.3**	**74.4**	175	6.3	**61.7**	**68.0**	67	16.4	**74.6**	**91.0**	p<0.001
**e330**	Support of people in positions of authority	244	11.5	**62.7**	**74.2**	176	11.4	**63.1**	**74.4**	68	11.8	**61.8**	**73.5**	ns
**e450**	Attitude of health professionals	244	9.4	**64.3**	**73.8**	176	7.4	**60.2**	**67.6**	68	14.7	**75.0**	**89.7**	p<0.001
**e460**	Societal attitudes	245	21.2	44.9	**65.7**	177	18.1	48.9	**66.9**	68	29.4	33.8	**63.2**	ns
**e585**	Education and training services, systems and policies	243	17.3	48.2	**65.4**	175	21.7	**54.3**	**76.0**	68	5.9	32.4	*38*.*2*	p<0.001
**e590**	Labour and employment services, systems and policies	244	30.3	29.1	**59.4**	176	34.9	29.0	**63.1**	68	20.6	29.4	**50.0**	ns
**e440**	Attitude of personal care providers and assistants	243	5.4	**53.5**	**58.9**	175	5.7	**54.3**	**60.0**	68	4.4	**51.4**	**55.9**	ns
**e465**	Social norms, practices and ideologies	245	14.3	44.5	**58.8**	177	10.2	**50.0**	**60.2**	68	25.0	30.9	**55.9**	ns
**e340**	Support of personal care providers and assistants	242	3.3	**55.4**	**58.7**	175	4.6	**60.0**	**64.6**	67	0.0	43.3	*43*.*3*	p<0.01
**e115**	Products and technology for personal use in daily living	244	8.2	46.3	**54.5**	176	8.5	39.2	*47*.*7*	68	7.4	**64.7**	**72.1**	p<0.001
**e1101**	Drugs	223	3.6	44.0	*47*.*5*	173	3.5	35.3	*38*.*7*	50	4.0	**74.0**	**78.0**	p<0.001
**e1100**	Food	239	6.3	34.0	*40*.*2*	172	7.6	24.4	*32*.*0*	67	3.0	**53.7**	**56.7**	p<0.001

* barr = barrier; facil = facilitator

**Table 10:** Frequency of impact of ICF categories of the extended ICF checklist for oral health for “Environment”, cited as a barrier by over 25% of participants in any country (excepting England due to small sample size).

	ICF items	ARGENTINA				FRANCE				IRELAND				SPAIN			
	Items cited by over 25% in any country	n	% barr*	% facil*	% impact	n	% barr*	% facil*	% impact	n	% barr*	% facil*	% impact	n	% barr*	% facil*	% impact
**e460**	Societal attitudes	61	4.9	**70.5**	**75.4**	60	40.0	3.3	43.3	59	25.4	39.0	**64.4**	60	13.3	**68.3**	**81.7**
**e465**	Social norms, practices and ideologies	61	11.5	**75.5**	**86.9**	60	5.0	5.0	10	59	27.1	33.9	**61.0**	60	15.0	**66.7**	**81.7**
**e540**	Transportation services, systems and policies	61	**73.7**	3.3	**77.1**	60	6.7	**75.0**	**81.7**	59	6.8	**78.0**	**84.8**	60	30.0	48.3	**78.3**
**e570**	Social security services, systems and policies	61	**86.9**	4.9	**91.8**	59	3.4	**94.9**	**98.3**	59	3.4	**81.4**	**84.8**	60	23.3	**55.0**	**78.3**
**e575**	General social support services, systems and policies	61	**73.8**	21.3	**95.1**	59	27.1	**67.8**	**94.9**	59	1.7	**78.0**	**79.7**	60	26.7	**50.0**	**76.7**
**e580**	Health services, systems and policies	61	**86.9**	1.6	**88.5**	60	16.7	**81.7**	**98.3**	59	11.9	**84.8**	**96.6**	60	23.3	**50.**	**73.3**
**e585**	Education and training services, systems and policies	61	19.7	**55.7**	**75.4**	59	17.0	**52.5**	**64.5**	58	0.0	36.2	36.2	60	33.3	46.7	**80.0**
**e590**	Labour and employment services, systems and policies	61	**75.4**	18.0	**93.4**	60	3.3	13.3	16.7	58	12.1	36.2	48.3	60	31. 7	45.0	**76.7**

* barr = barrier; facil = facilitator

#### ICF profile

A list of all 33 ICF items rated by over 50% of participants in both groups is given in Table 11. These items may be considered to be the common ICF profile for the study population.

**Table 11:** ICF items reported by >50% of participants in both the ‘Developmental disability’ and ‘Medically compromised’ groups (n = 33 items).

Impaired Body functions (n = 6)		% impact whole population
**b130**	Energy and drive function	78%
**b152**	Emotional function	76%
**b5102**	Chewing function	66%
**b5103**	Manipulation of food in the mouth	60%
**b5101**	Biting function	53%
**b735**	Muscle tone function	68%
**Impaired Oral structures (n = 2)**		
**s3200**	Structure of the teeth	73%
**s3201**	Structure of the gums	63%
**Restricted participation (n = 8)**		
**d230**	Carrying out daily routine	79%
**d240**	Handling stress / psychological demands	81%
**d570**	Looking after one's health	67%
**d770**	Intimate relationships	84%
**d845**	Acquiring, keeping and terminating a job	88%
**d870**	Economic self-sufficiency	79%
**d910**	Community life	78%
**d920**	Recreation and leisure including socialising	78%
**Impact of Environmental factors (n = 18)**		
**e310**	Support of immediate family	94%
**e320**	Support of friends	83%
**e330**	Support of people in positions of authority	74%
**e355**	Support of health professionals	74%
**e360**	Support of other professionals	69%
**e410**	Attitude of immediate family	92%
**e415**	Attitude of extended family	80%
**e420**	Attitude of friends	78%
**e440**	Attitude of personal care providers and assistants	59%
**e450**	Attitude of health professionals	74%
**e455**	Attitude of health-related professionals	73%
**e460**	Societal attitudes	66%
**e465**	Social norms, practices and ideologies	59%
**e540**	Transportation services, systems and policies	80%
**e570**	Social security services, systems and policies	87%
**e575**	General social support services, systems and policies	86%
**e580**	Health services, systems and policies	89%
**e590**	Labour and employment services, systems and policies	59%

## Discussion

The results of this study show that the ICF can be used to identify common factors of functioning, participation and environment amongst patients requiring Special care dentistry as defined by the International Association for Disability and Oral Health [1] (Table 11). The study also confirms a high level of oral disease and oral dysfunction in this population. These findings are important as they show that the ICF may provide insight into the underlying determinants of the inequality suffered by this group in terms of oral health. In particular, 33 items of the ICF were common to participants in both the ‘Developmental disability’ and ‘Medically compromised’ groups with a high prevalence of shared impact of environmental factors in relation to services, systems and policies.

The aim of recruiting a wide range of different patients presenting to specialist services was attained. The inclusion of those patients with the most complex needs and the most heterogeneous conditions was intentional and the sample was not designed to be representative. The aim was to explore all the domains of the ICF and to be as exhaustive as possible. In line with the study design, the population therefore presented a very high prevalence of different medical disorders. The heterogeneity of the sample was confirmed by the comparison of two arbitrary groups–those with ‘Developmental Disability’ and those ‘Medically Compromised’ patients.

Despite the high prevalence of medical conditions, the subjective general health of 83% of persons in the ‘Developmental disability’ group was rated as good or very good. This ‘disability paradox’, whereby persons with disability consider themselves to be in generally good health, has been reported elsewhere [17] and there have been calls for new measures of perceived health status that do not confound function with health [18]. Impaired mental functions were predominantly reported in the ‘Developmental disability’ group but it was interesting to note that eight of the mental function items were impaired for over a quarter of the ‘Medically compromised’ group, in particular ‘energy and drive’ and ‘emotional functions’. This was reflected in the subjective appraisals of mental health as being moderate to very poor for over a third of all participants.

Participants presented high DMFT scores, over 50% prevalence of impaired dental structures and a high incidence of dental disease (92%). Participants in the ‘Medically compromised’ group had a significantly higher DMFT and more missing teeth than those in the ‘Developmental disability’ group. However, this was to be expected with regards to the higher average age of the ‘Medically compromised’ group. In addition, 23% presented with less than five functional dental pairs, a marker of masticatory deficiency [16]. Almost half of participants did not perceive themselves as being in good oral health, although this finding was biased by the fact that they were attending dental services at the time of responding. What is most important is that the oral functions of ‘chewing’, ‘manipulating food in the mouth’, and ‘biting’ were impaired for the majority of participants, as was the participation item ‘eating’. Other oral function items were impaired at a level of over 25% and impaired swallowing affected a third of participants. Levels of oral dysfunction were higher for this adult population than in a previous similar study looking at children attending specialist services [19] and reflected data published in relation to older adults with functional disability [20]. This gives a picture of extremely high prevalence of oral disability that is likely to progressively exacerbate problems of general ill-health and developmental disability over time. When assimilated with the data reporting a low number of functional pairs, and a high number of missing and decayed teeth, it seems legitimate to sound the alarm regarding access to oral health promotion, prevention and treatment for this population.

Participation was particularly affected in the ‘Developmental disability’ group where 38 items were restricted for over 50% of participants. However, 29 items of participation were also restricted for over 25% of those in the ‘Medically compromised’ group. From those items that were highly restricted in both groups, it would seem that ‘Acquiring, keeping and terminating a job’, ‘Intimate relationships’, ‘Handling stress and psychological demands’, ‘Economic self-sufficiency’, ‘Carrying out daily routine’ and ‘Recreation and leisure’ are the most likely to be impacted by those requiring specialist dental care, regardless of medical diagnosis. These restrictions in participation were similar to those found in an ICF study comparing a range of different health conditions [21]. It is interesting to note that in an ICF study exploring the professional perspective of oral health [9] ‘Acquiring, keeping and terminating a job’, and ‘Carrying out daily routine’ did not make the list of ICF items considered relevant to oral health by professionals.

In terms of environment, most factors were rated as facilitators, particularly the support given by, and the attitudes of, family, friends and professionals (Table 9). This provides evidence that health care professionals can be instrumental in reducing inequalities in health. Certain environmental factors identified as barriers were related to ‘Services, systems and policies’ including, health, social security, general support, transportation, and labour services systems and policies. As shown in Table 10, this negative impact was particularly true in Argentina, but was echoed to a lesser extent in Spain. In the pre-cited study investigating professional opinion internationally, professionals did not rate transportation, and labour services systems and policies as being particularly relevant to oral health [9]. In addition, ‘Societal attitudes’ was the only item reported predominantly as a barrier for a population of children attending specialist dental services. This discrepancy may illustrate the societal assumption that an adult should be self-sufficient with regards their daily needs. Different barriers and facilitators thus emerge as children transition into adulthood, particularly in relation to public services.

When looking at the ‘ICF profile’ of the study group (Table 11), in addition to the problem of high prevalence of impaired oral health and function, the factors cited all relate to the social determinants of health at a population level [22,23]. Finding a job, attaining economic self-sufficiency, and participation in recreational and community activities are essential for maintaining a place within the social gradient and for the prevention of social exclusion. The ability to form and maintain intimate relationships is extremely important, especially when coupled with the positive influence of both practical and moral support from family and friends. Social support networks can help to combat problems with daily routine and health maintenance, thus reducing stress and risk of additional mental health problems such as depression. However, environmental barriers within the very services, systems and policies designed to relieve problems of participation and financial autonomy clearly need to be addressed. These results are compatible with other biopsychosocial models of health, such as that proposed by Wilson & Cleary [24], but have the advantage of being based on empirical findings and providing greater detail of specific influences. One way of reducing the impact of these social determinants on health is by the proactive identification and support of populations at risk of inequality and it is hoped that an ICF Core Set for Oral Health might be used in this context.

### Study limitations

This study was limited by the study population size and the fact that a convenience sample was used. However, the aim of the study was not to recruit a representative sample but to try and be as exhaustive as possible in terms of patient profile. This objective of recruiting a heterogeneous sample of patients from an international background was attained with a wide age range and a wide range of medical profiles, however it was impossible to check for data saturation without a second sample. It was unfortunate that data collection had to be discontinued in England. The ethical approval process was extremely lengthy at this centre and the investigator moved institutions before data collection could be completed. It was decided to include the English data in the analysis regardless of the small sample as the aim was not to compare countries but types of patients, and because the inclusion was compatible with the aim of exhaustivity.

### Conclusions and perspectives

Common aspects of functioning, participation and environment were found amongst a heterogeneous population of adults attending specialist dental services, along with a high prevalence of poor oral health and function.

WHO methodology requires four preliminary studies to inform a consensus process in the development of an ICF Core Set within a given discipline. This study represents the preliminary adult empirical study necessary for the development of an ICF Core Set in Oral Health. The other preliminary studies are either completed or underway [16,18,21]. Once established, the Core Set could be used to target populations in need of compensatory measures to combat inequality in oral health, but also as an outcome measure of both individual and population level interventions in oral health.

## Supporting information

S1 File Coding for the ICF Checklist for Oral Health. This is the coding guide for the ICF Checklist used in the study.
(PDF)

S2 File Minimal anonymised data set. Excel file containing minimal anonymised data set.
(XLSX)
