# Supplementary material for: Identifying common factors of functioning, participation and environment amongst adults requiring specialist oral health care using the International Classification of Functioning, disability and health
Source: PLoS One. 2018 Jul 3;13(7):e0199781. doi: 10.1371/journal.pone.0199781 (PMC6029782; doi:10.1371/journal.pone.0199781)
Supplement: S1 File — This is the coding guide for the ICF Checklist used in the study. (PDF) [file pone.0199781.s001.pdf]

CODING THE MODIFIED ICF CHECKLIST – SPECIAL CARE DENTISTRY

H1. Data Source *Please circle the information used for compiling this report*:

1) written records **2)** primary respondent **3)** parent or guardian

4) other informant **5)** direct observation

H2. Date of data collection __ __ / __ __ / __ __

H3. Investigator number __ __

DEMOGRAPHIC INFORMATION

A1. Family Name (3 initials) __ __ __ **First Name (2 initials)** __ __

A2. Sex female (2) male (1)

A3. Date of Birth __ __ / __ __ / __ __

A4. Primary Residence

1 Own home
2 Caregiver's home
3 Institution
4 Hospital

A5. Current occupation

1 Mainstream employment
2 Protected employment
3 Unpaid work
4 Student
5 Unemployed (medical condition)
6 Unemployed (incapacity)
7 Retired
8 Home-maker
9 Other ________________

A6. Medical diagnosis (e.g. Down syndrome)

No medical condition *give ICD codes*

_________________________________ ICD Code ________________

_________________________________ ICD Code ________________

_________________________________ ICD Code ________________

_________________________________ ICD Code ________________

_________________________________ ICD Code ________________

Unspecified or undiagnosed health condition ____

Please complete appendix I "ICD Main categories" if diagnosis unclear.

A7. Oral diagnosis (e.g. caries)

No oral condition | *give ICD-DA codes*

___________________________________ ICD-DA Code ________________

___________________________________ ICD-DA Code ________________

___________________________________ ICD-DA Code ________________

___________________________________ ICD-DA Code ________________

___________________________________ ICD-DA Code ________________

Unspecified or undiagnosed oral condition ____

Please complete appendix II "ICD-DA Main categories" if diagnosis unclear.

A8. Number of teeth __ __

A9. Number of functional pairs of teeth __ __

Number of pairs of upper and lower premolars and molars with occlusal contact, when wearing dentures if appropriate.

A10. Number of decayed teeth (D) __ __

Number of missing teeth (M) __ __

Number of filled teeth (F) __ __

TOTAL DMFT __ __

Decay refers to visible, cavitated dentinal caries lesions. Missing refers to missing due to extraction for dental disease.

BRIEF HEALTH INFORMATION

Please circle the correct reply and complete details where necessary:

B1. Data Source

1) Self report **2)** Parent or caregiver report **3)** Clinician administered

B2. Height __ __ __ cm or __ feet __ __ inches

B3. Weight __ __ __ kg or __ __ stones __ __ pounds

B4. How do you rate your **physical health** in the past month?

Very good Good Moderate Bad Very bad

B5. How do you rate your **mental and emotional health** in the past month?

Very good Good Moderate Bad Very bad

B6. How would you rate your overall **oral health?**

Very good Good Moderate Bad Very bad

B7. Do you currently have any **disease(s) or disorder(s)?**

No Yes: *please specify*___________________________________

____

B8. Have you been **hospitalised** in the last year?

No Yes: *give reason* ___________________________________

for how long? _________ days

B9. Are you taking any **medication?** (either prescribed or over the counter)

No Yes: *please specify* ___________________________________

____

B10. Do you **smoke?**

No Yes: *type and daily quantity*:___________________________

B11. Do you consume **alcohol** or **drugs?**

No Yes: *type and daily quantity*:___________________________

B12. Do you use any **assistive devices** such as glasses, hearing aid, wheelchair etc.?

No Yes: *please specify* ___________________________________

____

B13. Does **anybody assist you** with your self-care, shopping or daily activities?

No Yes: *please specify* ___________________________________

____

B14. Are you receiving any kind of **treatment for your health** such as physiotherapy**?**

No Yes: *please specify* ___________________________________

____

B15. Additional significant information on your **past or present health?**

____

____

B16. IN THE PAST MONTH have you **cut back** on your **usual activities** or work because of your health condition?

No Yes: *please specify* ___________________________________

B17. IN THE PAST MONTH have you been **totally unable to carry out your usual activities** or work because of your health condition?

No Yes: *please specify*___________________________________

PART 1: BODY FUNCTIONS

Body Functions are the physiological functions of body systems.

Impairments are problems in body function as a significant deviation or loss.

Qualifiers:

0 ***No impairment*** means the person has no problem
1 ***Mild impairment*** means a problem that is present less than 25% of the time, with an intensity which is tolerable and which happens rarely over the last 30 days
2 ***Moderate impairment*** means that a problem is present less than 50% of the time, with an intensity which interferes in day to day life and which happens occasionally over the last 30 days
3 ***Severe impairment*** means that a problem is present more than 50% of the time, with an intensity which partially disrupts day to day life and which happens frequently over the last 30 days
4 ***Complete impairment*** means that a problem is present over 95% of the time, with an intensity that totally disrupts day to day life and happens every day over the last 30 days.
8 ***Not specified*** means there is insufficient information to specify the severity of the impairment
9 ***Not applicable*** means it is inappropriate (e.g. menstruation functions for males)

ITEMS RELATING DIRECTLY TO THE ORAL SPHERE	
**Body Function**	**Qualifier**
**b250** Taste	0 1 2 3 4 8 9
**b510**0 Sucking	0 1 2 3 4 8 9
**b5101** Biting (*front teeth*)	0 1 2 3 4 8 9
**b510**2 Chewing (*back teeth*)	0 1 2 3 4 8 9
**b5103** Manipulation of food in the mouth	0 1 2 3 4 8 9
**b5104** Salivation	0 1 2 3 4 8 9
**b5105** Swallowing	0 1 2 3 4 8 9
**b5106** Regurgitation function (*vomiting*)	0 1 2 3 4 8 9

ITEMS RELATING DIRECTLY TO FUNCTION HAVING A DIRECT OR INDIRECT INFLUENCE ON ORAL HEALTH	
**Body Function**	**Qualifier**
**b110** Consciousness	0 1 2 3 4 8 9
**b114** Orientation (*time, place, person)*	0 1 2 3 4 8 9
**b117** Intellectual (*incl. retardation, dementia*)	0 1 2 3 4 8 9
**b122** Global psychosocial functions (*incl. social interaction*)	0 1 2 3 4 8 9
**b130** Energy and drive functions	0 1 2 3 4 8 9
**b140** Attention	0 1 2 3 4 8 9
**b144** Memory	0 1 2 3 4 8 9

**b147** Psychomotor functions *(e.g. control when excited)*	0 1 2 3 4 8 9
**b152** Emotional functions (*appropriate emotion, control*)	0 1 2 3 4 8 9
**b156** Perceptual functions	0 1 2 3 4 8 9
**b164** Higher level cognitive functions (*problem solving*)	0 1 2 3 4 8 9
**b167** Language (*recognition and use of*)	0 1 2 3 4 8 9
**b180** Experience of self and time (*incl. body image*)	0 1 2 3 4 8 9
**b210** Seeing	0 1 2 3 4 8 9
**b230** Hearing	0 1 2 3 4 8 9
**b255** Smell	0 1 2 3 4 8 9
**b260** Proprioceptive function (*relative position of body parts)*	0 1 2 3 4 8 9
**b265** Touch function	0 1 2 3 4 8 9
**b270** Sensory functions (*temperature, vibration, pressure…)*	0 1 2 3 4 8 9
**b280** Sensation of pain	0 1 2 3 4 8 9
**b310** Voice functions	0 1 2 3 4 8 9
**b320** Articulation function	0 1 2 3 4 8 9
**b410** Heart	0 1 2 3 4 8 9
**b430** Haematological (*blood*)	0 1 2 3 4 8 9
**b435** Immunological (*allergies, hypersensitivity)*	0 1 2 3 4 8 9
**b440** Respiration (*breathing)*	0 1 2 3 4 8 9
**b450** Additional respiratory functions (*coughing, yawning…*)	0 1 2 3 4 8 9
**b515** Digestive functions	0 1 2 3 4 8 9
**b530** Weight maintenance functions	0 1 2 3 4 8 9
**b540** General metabolic functions	0 1 2 3 4 8 9
**b710** Mobility of joints functions	0 1 2 3 4 8 9
**b730** Muscle power	0 1 2 3 4 8 9
**b735** Muscle tone	0 1 2 3 4 8 9
**b760** Control of voluntary movements	0 1 2 3 4 8 9
**b765** Involuntary movements	0 1 2 3 4 8 9
**b810** Protective functions of the skin	0 1 2 3 4 8 9
**ANY OTHER BODY FUNCTIONS**	
	0 1 2 3 4 8 9
	0 1 2 3 4 8 9

PART 2: BODY STRUCTURES

Body Structures are anatomical parts of the body such as organs, limbs and their components.

Impairments are problems in structure as a significant deviation or loss.

First qualifier: *Extent of impairment*
**0** ***No impairment*** means the person has no problem<br>**1** ***Mild impairment*** means a problem is present less than 25% of the time, with an intensity which is tolerable and which happens rarely over the last 30 days<br>**2** ***Moderate impairment*** means that a problem is present less than 50% of the time, with an intensity which interferes in day-to-day life and which happens occasionally over the last 30 days<br>**3** ***Severe impairment*** means that a problem is present more than 50% of the time, with an intensity which partially disrupts day to day life and which happens frequently over the last 30 days<br>**4** ***Complete impairment*** means that a problem is present over 95% of the time, with an intensity that totally disrupts day to day life and happens every day over the last 30 days<br>**8** ***Not specified*** means that there is insufficient information to specify the severity of the impairment<br>**9** ***Not applicable*** means it is inappropriate (e.g. menstruation functions for males)

ITEMS RELATING DIRECTLY TO THE ORAL SPHERE	
**Body Structure**	**First Qualifier**
**s3200** Teeth	0 1 2 3 4 8 9
**s3201** Gums	0 1 2 3 4 8 9
**s3202** Palate	0 1 2 3 4 8 9
**s3203** Tongue	0 1 2 3 4 8 9
**s3204** Lip	0 1 2 3 4 8 9

PART 3: ACTIVITY AND PARTICIPATION

Activity limitations and participation restrictions

Activity is the execution of a task or action by an individual.
Activity limitations are difficulties an individual may have in executing activities.
Participation is involvement in a life situation.
Participation restrictions are problems an individual may have in involvement in life situations.

The **performance qualifier** indicates the *extent of participation restriction* by describing the persons *actual performance* of a task or action *in his or her current environment.* Because the current environment brings in the societal context, performance can also be understood as "involvement in a life situation" or "the lived experience" of people in the actual context in which they live. This context includes the environmental factors – all aspects of the physical, social and attitudinal world that can be coded using the Environmental codes. The performance qualifier measures the difficulty the respondent experiences in *doing things, assuming they want to do them.*

The **capacity qualifier** indicates the *extent of activity limitation* by describing the *person's ability* to execute a task or an action. The capacity qualifier focuses on limitations that are inherent or intrinsic features of the person themselves. These limitations should be direct manifestations of the respondent's health state, *without assistance.* By assistance we mean the help of another person, or assistance provided by an adapted or specially designed tool or vehicle, or any form of environmental modification to a room, home, workplace etc. The level of capacity should be judged relative to that normally expected of the person, or the person's capacity before they acquired their health condition.

General guidelines for participation and activities.

The following probes are proposed as a guide to help the examiner when interviewing the respondent about problems in functioning and life activities, in terms of the distinction between capacity and performance. Take into account all personal information known about the respondent and ask any additional probes as necessary. Probes should be rephrased as open-ended questions if necessary to elicit greater information.
Under each domain there are two kinds of probes:
The first probe tries to get the respondent to focus on his or her ***capacity*** to do a task or action, and in particular to focus on limitations in capacity that are ***inherent or intrinsic features*** of the person themselves. These limitations should be direct manifestations of the respondent's health state without assistance. By ***assistance*** we mean the help of another person, or assistance provided by an adapted or specially designed tool or vehicle, or any form of environmental modification to a room, home, workplace and so on. The level of capacity should be judged relative to that normally expected of the person, or the person's capacity before they acquired their health condition.
The second probe focuses on the respondent's ***actual performance*** of a task or action in the person's actual situation or surroundings, and elicits information about the effects of environmental barriers or facilitators. It is important to emphasise that you are only interested in the extent of difficulty the respondent has in doing things ***assuming that they want to do them.*** Not doing something is irrelevant if the person chooses not to do it.

Example: Self Care

(Capacity)

1) In your present state of health, how much difficulty do you have washing yourself without assistance?
2) How does this compare with someone, just like you only without your health condition?

(Performance)

1) In your own home, how much of a problem do you actually have washing yourself?
2) Is this problem made worse, or better, by the way your home is set up or the specially adapted tools that you use?
3) Is your capacity to wash yourself without assistance more or less than what you actually do in your present surroundings?

<table>
<tr><td>First Qualifier: Performance in current environment
Extent of performance restriction</td><td>Second Qualifier: Capacity without assistance
Extent of activity limitation</td></tr>
<tr><td colspan="2">0 No difficulty means the person has no problem.
1 Mild difficulty means a problem that is present less than 25% of the time, with an intensity which is tolerable and which happens rarely over the last 30 days.
2 Moderate difficulty means that a problem is present less than 50% of the time, with an intensity which interferes in day to day life and which happens occasionally over the last 30 days.
3 Severe difficulty means that a problem is present more than 50% of the time, with an intensity which partially disrupts day to day life and which happens frequently over the last 30 days.
4 Complete difficulty means that a problem is present over 95% of the time, with an intensity that totally disrupts day to day life and happens every day over the last 30 days.
8 Not specified means there is insufficient information to specify the severity of the difficulty.
9 Not applicable means it is inappropriate (e.g. menstruation functions for males).</td></tr>
</table>

ITEMS RELATING DIRECTLY TO FUNCTION HAVING A DIRECT OR INDIRECT INFLUENCE ON ORAL HEALTH		
**Activities and participation**	**Performance Qualifier**	**Capacity Qualifier**
**d110** Watching	0 1 2 3 4 8 9	0 1 2 3 4 8 9
**d115** Listening	0 1 2 3 4 8 9	0 1 2 3 4 8 9
**d120** Purposeful sensing	0 1 2 3 4 8 9	0 1 2 3 4 8 9
**d130** Copying	0 1 2 3 4 8 9	0 1 2 3 4 8 9
**d155** Acquiring skills	0 1 2 3 4 8 9	0 1 2 3 4 8 9
**d163** Purposeful thinking	0 1 2 3 4 8 9	0 1 2 3 4 8 9
**d175** Solving problems	0 1 2 3 4 8 9	0 1 2 3 4 8 9
**d177** Making decisions	0 1 2 3 4 8 9	0 1 2 3 4 8 9
**d210** Undertaking a single task	0 1 2 3 4 8 9	0 1 2 3 4 8 9
**d220** Undertaking multiple tasks	0 1 2 3 4 8 9	0 1 2 3 4 8 9
**d230** Carrying out daily routine	0 1 2 3 4 8 9	0 1 2 3 4 8 9
**d240** Handling stress and other psychological demands	0 1 2 3 4 8 9	0 1 2 3 4 8 9
**d310** Communicating with -- receiving -- spoken messages	0 1 2 3 4 8 9	0 1 2 3 4 8 9
**d315** Communicating with -- receiving -- non-verbal messages	0 1 2 3 4 8 9	0 1 2 3 4 8 9
**d330** Speaking	0 1 2 3 4 8 9	0 1 2 3 4 8 9
**d335** Producing non-verbal messages	0 1 2 3 4 8 9	0 1 2 3 4 8 9
**d350** Conversation	0 1 2 3 4 8 9	0 1 2 3 4 8 9
**d410** Changing basic body position	0 1 2 3 4 8 9	0 1 2 3 4 8 9
**d415** Maintaining a body position	0 1 2 3 4 8 9	0 1 2 3 4 8 9
**d420** Transferring oneself	0 1 2 3 4 8 9	0 1 2 3 4 8 9
**d430** Lifting and carrying objects	0 1 2 3 4 8 9	0 1 2 3 4 8 9
**d440** Fine hand use (*picking up, grasping)*	0 1 2 3 4 8 9	0 1 2 3 4 8 9
**d445** Hand and arm use (*pulling, reaching, turning)*	0 1 2 3 4 8 9	0 1 2 3 4 8 9
**d510** Washing oneself (*bathing, drying, washing hands etc)*	0 1 2 3 4 8 9	0 1 2 3 4 8 9
**d520** Caring for body parts (*brushing teeth, shaving, grooming etc)*	0 1 2 3 4 8 9	0 1 2 3 4 8 9
**d550** Eating	0 1 2 3 4 8 9	0 1 2 3 4 8 9
**d560** Drinking	0 1 2 3 4 8 9	0 1 2 3 4 8 9

**d570** Looking after one's health (*maintaining diet and fitness, following medical advice…)*	0 1 2 3 4 8 9	0 1 2 3 4 8 9
**d620** Acquisition of goods and services (*shopping etc)*	0 1 2 3 4 8 9	0 1 2 3 4 8 9
**d630** Preparation of meals (*cooking etc)*	0 1 2 3 4 8 9	0 1 2 3 4 8 9
**d710** Basic interpersonal interactions	0 1 2 3 4 8 9	0 1 2 3 4 8 9
**d720** Complex interpersonal interactions (*forming relationships*)	0 1 2 3 4 8 9	0 1 2 3 4 8 9
**d730** Relating with strangers	0 1 2 3 4 8 9	0 1 2 3 4 8 9
**d740** Formal relationships	0 1 2 3 4 8 9	0 1 2 3 4 8 9
**d770** Intimate relationships (*incl. sexual*)	0 1 2 3 4 8 9	0 1 2 3 4 8 9
**d820** School education	0 1 2 3 4 8 9	0 1 2 3 4 8 9
**d845** Acquiring, keeping and terminating a job	0 1 2 3 4 8 9	0 1 2 3 4 8 9
**d860** Basic economic transactions	0 1 2 3 4 8 9	0 1 2 3 4 8 9
**d870** Economic self-sufficiency	0 1 2 3 4 8 9	0 1 2 3 4 8 9
**d910** Community life (*clubs, associations, ceremonies*)	0 1 2 3 4 8 9	0 1 2 3 4 8 9
**d920** Recreation and leisure *(including socialising)*	0 1 2 3 4 8 9	0 1 2 3 4 8 9
**d950** Political life and citizenship (*engaging as a citizen, voting*)	0 1 2 3 4 8 9	0 1 2 3 4 8 9
**ANY OTHER ACTIVITIES OR PARTICIPATION**		
	0 1 2 3 4 8 9	0 1 2 3 4 8 9
	0 1 2 3 4 8 9	0 1 2 3 4 8 9

PART 4: ENVIRONMENTAL FACTORS

Environmental factors make up the physical, social and attitudinal environment in which people live and conduct their lives.

**Qualifier in environment:**	**0** No barriers	**0** No facilitator
**Barriers or facilitators**	**1** Mild barriers	+**1** Mild facilitators
	**2** Moderate barriers	+**2** Moderate facilitators
	**3** Severe barriers	**+3** Substantial facilitators
	**4** Complete barriers	**+4** Complete facilitators

ITEMS RELATING TO ENVIRONMENTAL FACTORS HAVING A DIRECT OR INDIRECT INFLUENCE ON ORAL HEALTH	
**Environmental factor**	**Barrier or facilitator**
**e1 Products and technology**	
**e1100** Is appropriate food available to you?	4 3 2 1 0 +1 +2 +3 +4
**e1101** Are appropriate drugs available to you?	
**e115** Are appropriate products or aids for personal use in daily living available to you?	4 3 2 1 0 +1 +2 +3 +4
**e125** Are appropriate products or aids for communication available to you?	4 3 2 1 0 +1 +2 +3 +4
**e3 Support and relationships**	
**e310** Are immediate family supportive of you?	4 3 2 1 0 +1 +2 +3 +4
**e320** Are friends supportive of you?	4 3 2 1 0 +1 +2 +3 +4
**e330** Are people in position of authority supportive of you?	4 3 2 1 0 +1 +2 +3 +4
**e340** Are personal care providers and personal assistants supportive of you?	4 3 2 1 0 +1 +2 +3 +4
**e355** Are health professionals supportive of you?	4 3 2 1 0 +1 +2 +3 +4
**e360** Are health related professionals supportive of you?	4 3 2 1 0 +1 +2 +3 +4

**e4 Attitudes**	
**e410** Are individual attitudes of immediate family members favourable to you?	4 3 2 1 0 +1 +2 +3 +4
**e415** Are individual attitudes of extended family members favourable to you?	4 3 2 1 0 +1 +2 +3 +4
**e420** Are individual attitudes of friends favourable to you?	4 3 2 1 0 +1 +2 +3 +4
**e440** Are individual attitudes of personal care providers and personal assistants favourable to you?	4 3 2 1 0 +1 +2 +3 +4
**e450** Are individual attitudes of health professionals favourable to you?	4 3 2 1 0 +1 +2 +3 +4
**e455** Are individual attitudes of health related professionals favourable to you?	4 3 2 1 0 +1 +2 +3 +4
**e460** Are societal attitudes favourable to you? *(i.e. the opinions and beliefs generally held by people in your culture, society or social group)*	4 3 2 1 0 +1 +2 +3 +4
**e465** Are social norms, practices and ideologies favourable to you? *(i.e. common customs, practices or behaviours within your social context)*	4 3 2 1 0 +1 +2 +3 +4
**e5 Services, systems and policies**	
**e540** Are appropriate transportation services, systems and policies available to you?	4 3 2 1 0 +1 +2 +3 +4
**e570** Are appropriate social security services, systems and policies available to you?	4 3 2 1 0 +1 +2 +3 +4
**e575** Are general social support services, systems and policies available to you?	4 3 2 1 0 +1 +2 +3 +4
**e580** Are appropriate health services, systems and policies available to you?	4 3 2 1 0 +1 +2 +3 +4
**e585** Are appropriate education services, systems and policies available to you?	4 3 2 1 0 +1 +2 +3 +4
**e590** Are appropriate labour and employment services, systems and policies available to you?	4 3 2 1 0 +1 +2 +3 +4
**ANY OTHER ENVIRONMENTAL FACTORS**	
	4 3 2 1 0 +1 +2 +3 +4
	4 3 2 1 0 +1 +2 +3 +4
